# Book Notices

**Published:** 1892-10

**Authors:** 


PYE'S SURGICAL HANDICRAFT, a *Manual of Surgical Manipulations*, minor surgery, and other matters connected with the work of house surgeons and surgical dressers. 300 illustrations on wood. First American, from 3rd London edition, revised and edited by J. H. R. Crowle, F. R. C. S., Surgical Registrar to St. Mary's Hospital, Surgical Tutor and Joint Lecturer on Practical Surgery in the Medical School. Complete in one volume. New York: E. B. Treat, Publisher, 5 Cooper Union. 1892. Bound in sheep. 594 pages. Price, $4.

The average physician doing a general practice, as by far the large majority are compelled to do, pays too little attention to the mechanical part of his profession, the *art* of surgery. He may understand thoroughly, the principles of surgery, and yet be unable to introduce a catheter skillfully, or apply a bandage properly. We have seen an eminent professor of surgery sweat over the attempt to introduce a male catheter, and his student step up and introduce it for him with dexterity. The student had begun on the ground. A work like *Pye's Hand-book* is an

*essential* to every student of medicine; and the oldest practitioner would do well to stop long enough to go through it carefully and to put in practice the instructions as to bandaging, etc., as he goes along. The work is well known and has been a favorite for some years. The volume before us is a revision of the English editions, and is the first issued from the American press. It is indeed a *hand-book*, small and compact, strongly put together, and can be easily handled. Printed in clear Ronaldson type, easily read, and on clean, strong, white paper. Treat, the publisher, excels in that feature of his works; they do not fall to pieces from handling, like many larger works do, and vex a person. We are much pleased with this book and can recommend it heartily.

---

NOSE AND THROAT, a Treatise on Disease of, in two volumes, by Francke Huntington Bosworth, A. M., M. D., Professor of Diseases of the Throat, in Bellevue Hospital Med. College, N. Y., Consulting Laryngologist to the Presbyterian Hospital, Consulting Physician to the Out-door Department Bellevue, etc. Vol. II. DISEASES OF THE THROAT. Three colored plates and 125 wood cuts. N. Y., 1892. Wm. Wood & Co. Bound in cloth. 830 pages. Price, cloth, net $6.00.

This work is one of a series ot works on "Specialties in the Practice of Medicine" being issued from the house of Wm. Wood & Co., the greet American medical publishers. The JOURNAL is in receipt of Vol. II, Diseases of the Throat. Vol. I was noticed in the JOURNAL when it first appeared; and we have but little to add to what was then said. Oculists especially, and practitioners in general, who are interested in the diseases of the nose and throat,—and all country and village physicians are compelled to treat this class of ailments more or less, will find Dr. Bosworth's book very satisfactory. It is full and exhaustive, giving the most minute details both of symptomatology and the technique of treatment. The trouble with most authors on special subjects is, they apparently assume that every reader is informed on details or fundamental principles, and omit all mention of things that it is essential to know;—in the fullness of their information they forget that all doctors are not *educated;* and a text-book, for such we take any authoritative work on any specialty to be, should make everything clear; should prepare the reader to understand what is going to be said on treatment. This we take to be Dr. Bosworth's idea,—for he is certainly explicit and minute. As a book for posting one's self, this work is superior. The illustrations are good, and the mechanical, or book-maker's part, is fully up to Wood & Co.'s high standard.

THE INTERNATIONAL MEDICAL ANNUAL and Practitioner's Index for 1892. Edited by P. W. Williams, M. D., Secretary of staff, assisted by a corps of thirty-two collaborators—European and American—specialists in their several departments. 644 octavo pages. Illustrated. $2.75. E. B. Treat, publisher, 5 Cooper Union, New York.

The tenth yearly issue of this valuable one-volume reference work is to hand; and it richly deserves and perpetuates the enviable reputation which its predecessors have made, for selection of material, accuracy of statement and great usefulness. The corps of department editors is representative in every respect. Numerous illustrations—many of which are in colors—make the "Annual" more than ever welcome to the profession, as providing, at a reasonable outlay, the handiest and best resume of medical progress yet offered.

Part one comprises the New Remedies, together with an extended Review of the Therapeutic Progress of the Year.

Part second, comprising the major portion of the book, is given to the consideration of treament; and is a retrospect of the year's work, with numerous original articles by eminent authorities.

The third—and last part—is made up of miscellaneous articles, such as Recent Advances in Bacteriology; Medical Photography; Sanitary Science; Use of Suppositories in the Treatment of Disease; Improvements in Pharmacy; New Inventions in Instruments and Appliances; Books of the Year, etc.

The arrangement of the work is alphabetical, and with its complete index, makes it a reference book of rare worth.

In short, the "Annual" is what it claims to be—a recapitulation of the year's progress in medicine, serving to keep the practitioner abreast of the times with reference to the medical literature of the world. Price, the same as in previous years.—$2.75.

